# Hair-Dye-Related Accidental Poisoning and Death

**DOI:** 10.7759/cureus.14607

**Published:** 2021-04-21

**Authors:** Niraj R Gowda, Joseph Delio, Amira Elshikh, Rahul Khosla

**Affiliations:** 1 Internal Medicine, George Washington University School of Medicine and Health Sciences, Washington, USA; 2 Pulmonary and Critical Care Medicine, George Washington University School of Medicine and Health Sciences, Washington, USA; 3 Pulmonary and Critical Care Medicine, Veterans Affairs Medical Center, Washington, USA

**Keywords:** para-phenylenediamine, rhabdomyolysis, acute renal failure, angioedema

## Abstract

Para phenylenediamine (PPD) is a common component of hair dye as well as temporary tattoos and is a well-known cause of type 4 hypersensitivity reactions from topical exposure. While there have been several cases reported in the literature describing toxicities following ingestion, there are a paucity of reports of severe systemic disease following topical exposure. Cases of PPD ingestion have been reported to present with angioedema-like reactions, often progressing to rhabdomyolysis and renal failure. To our knowledge, there have only been two reported cases of severe reactions following topical exposure to PPD. We present a case of a 59-year-old man with topical exposure to hair dye who presented with an angioedema-like reaction shortly after topical exposure to PPD containing hair dye that rapidly progressed to rhabdomyolysis, renal failure, and eventually death.

## Introduction

Para phenylenediamine (PPD) is a common component of hair dye and henna tattoos and is known to be associated with type 4 hypersensitivity reactions [1]. Although these reactions are rarely ever life-threatening, there have been several reported cases of PPD ingestion and poisoning that have led to acute renal failure and rhabdomyolysis [2-6]. To our knowledge, there have only been two reported cases of severe systemic toxicities following topical PPD exposure that included anaphylaxis and/or cardiac complications [7,8]. Our case describes a patient with topical PPD exposure with a clinical course typical of PPD ingestion with complications including rhabdomyolysis, renal failure requiring hemodialysis, and severe electrolyte derangements eventually leading to the patient’s demise. Currently, no known antidote exists for severe systemic PPD reactions. Prompt recognition and supportive therapy are mainstays of management.

## Case presentation

A 59-year-old man with a past medical history of atrial flutter status-post ablation, hypertension, osteoarthritis, and depression presented to the emergency department (ED) with a three-day history of a burning, erythematous rash involving the neck, forehead, and cheeks. He denied any history of allergies or taking new medications. He did report applying a new hair dye to his scalp and beard. No other environmental exposures were identified. Apart from an elevated blood pressure, all other vital signs were within normal limits. He was diagnosed with allergic contact dermatitis and was discharged home with a Solu-Medrol dose pack, ranitidine, loratadine, and instructions to return to the ED if symptoms worsened. 

Six days later, he returned to the ED with worsening asymmetric facial swelling (right > left), difficulty breathing, dysphagia with upper and lower lip swelling, and an erythematous rash with wheals on his bilateral upper extremities and neck. Labs were notable for a leukocytosis of 16.5 cell/L with an unremarkable basic metabolic panel and venous blood gas. Bedside laryngoscopy was performed by ENT showed normal mucosa, no pooling of secretions, and no laryngeal edema. He was admitted for angioedema and started on dexamethasone, famotidine, diphenhydramine. His lisinopril was discontinued out of an abundance of caution in the setting of angioedema of unknown etiology. On hospital day 1, he became febrile, tachycardic, and tachypneic with hypoxia. Repeat laryngoscope examination showed the increased base of tongue fullness, pooling of thick clear secretions, and laryngeal edema.

He was naso-tracheally intubated for airway protection and was started on broad spectrum antibiotics, including vancomycin and cefepime for possible sepsis. A computed tomography scan of his neck showed extensive soft tissue swelling and edema with extension to the anterolateral neck. Dexamethasone, famotidine, and diphenhydramine were continued during his entire. Blood cultures grew methicillin-resistant Staphylococcus aureus (MRSA) in two bottles and his antibiotics were narrowed to vancomycin. He was started on continuous renal replacement therapy (CRRT) for worsening renal failure. Dermatology was consulted for possible PPD allergy from recent hair dye use. PPD patch test was not performed as he was on systemic steroids and the yield was thought to be insignificant. On hospital day 3, he developed upper and lower lip mucosal lesions with necrotic erosions and overlying hemorrhagic crust. He was started on Valtrex for presumed (and eventually confirmed) herpes simplex virus 1 (HSV-1) infection. On hospital day 5, he showed significant clinical improvement with discontinuation of vasopressors and transition to intermittent hemodialysis. However, on hospital day 6, he became febrile to 107 F with a worsening metabolic profile including refractory hyperkalemia with prolonged PR and QRS intervals unresponsive to medical therapy and CRRT. His creatine phosphokinase was elevated to >22,000 units/L, and he developed bullous lesions with serous drainage of his right lower extremity as well as erythema and induration of his left axilla. Given his clinical deterioration and concern for compartment syndrome, emergent bedside fasciotomy was performed with no evidence of necrotic muscle. Shortly after the procedure, the patient went into cardiac arrest and expired.

## Discussion

PPD has been a common component of hair dye and henna tattoos for more than 100 years. There is a well-described PPD hypersensitivity reaction that typically presents as a type 4 hypersensitivity reaction after topical exposure that involves pruritus, erythema, and papules involving areas of dye exposure [1]. These reactions are rarely life-threatening and are typically self-limited; however, if treatment is pursued, the reaction is managed with topical and/or systemic steroids until improvement in symptoms. There is a growing incidence of PPD poisoning either through accidental or intentional ingestion reported in Africa and Asia as it is a major component in henna, an ink commonly used in these regions to temporarily color the hands, feet, and hair [2]. A retrospective review of 150 cases over 10 years of accidental or intentional PPD ingestion in Khartoum, Sudan found that 60% of the patients experienced acute renal failure requiring dialysis [3]. This renal failure is thought to be multifactorial secondary to the direct nephrotoxic effects of the compound itself and the compound’s induction of rhabdomyolysis and its subsequent heme-pigment induced nephropathy [3-6]. These patients also developed rapid-onset, severe edema of the face, tongue, neck, and larynx resulting in dyspnea often necessitating intubation to maintain the patient’s airway [3-4,6]. The progression of symptoms typically begins with facial swelling involving the larynx and it eventually progresses to rhabdomyolysis and subsequent renal failure as described in our patient [3-4,6].

There have only been two reported cases of severe reaction to topical application of PPD. In both of these cases, the patients experienced severe anaphylaxis and died. The first patient received typical treatment for anaphylaxis but, despite this, died [7]. In the second case, the patient experienced a myocardial infarction shortly after admission and subsequently died [8]. To our knowledge, our patient is the first reported case of PPD topical exposure following a typical clinical course of PPD ingestion. Our patient’s rapid progression from an angioedema-like presentation to rhabdomyolysis and subsequent acute renal failure requiring hemodialysis is similar to previously described courses of ingestion [2-6].

Currently, there is no specific antidote or treatment for patients suffering from either topical or ingested PPD poisoning. Airway management is key, especially in those suspected of having laryngeal edema. A rapid evaluation with laryngoscopy along with prompt intubation can decrease mortality. Prompt initiation of steroids and antihistamines to target mast cell degranulation is key as PPD poisoning is thought to be a type 1 hypersensitivity unlike the type 4 hypersensitivity reaction associated with topical use. Rhabdomyolysis as a direct effect of PPD-induced necrosis of skeletal muscle can be managed with aggressive hydration, and dialysis should be considered if the patient is believed to be experiencing heme-pigment induced acute renal failure [9,10]. Plasma exchange transfusion has been used with success for intoxication with Lawsone, an active ingredient in henna. Such methods could present a new avenue of therapy for PPD poisoning with the assumption that PPD is located in the circulating plasma and could be effectively removed [11].

## Conclusions

Prompt recognition of the diagnosis of PPD toxicity through history is essential. As there are no evidence-based treatments available, supportive care as outlined above remains the mainstay of treatment. Plasma exchange transfusion can be considered a possible last-line therapy for patients who fail to respond to supportive care. Our patient’s presentation is incredibly unique in that topical exposure to PPD presented like ingestion of PPD.
